# Bacterial Isolates and Antibacterial Resistance Patterns in a Patient with Acute Exacerbation of Chronic Obstructive Pulmonary Disease in a Tertiary Teaching Hospital, Southwest Ethiopia

**DOI:** 10.1155/2022/9709253

**Published:** 2022-08-31

**Authors:** Abdulhakim Mussema, Getenet Beyene, Mulatu Gashaw

**Affiliations:** ^1^School of Medical Laboratory Science, Jimma University, Jimma, Ethiopia; ^2^Department of Medical Laboratory Science, Wachemo University, Hosanna, Ethiopia

## Abstract

**Introduction:**

Chronic obstructive pulmonary disease (COPD) is a major cause of chronic morbidity and mortality worldwide. The natural course of COPD is characterized by acute exacerbation. Infectious agents, particularly bacteria, cause exacerbation of COPD in the majority. This study aimed to determine the bacteriology and antibiotic resistance patterns among patients with acute exacerbations of COPD (AECOPD) at Jimma Medical Center.

**Methods:**

A cross-sectional study was conducted from March to October 2019. Demographic, clinical, and sputa samples were collected from 39 study participants, who were diagnosed with AECOPD. Bacterial pathogens were identified using standard bacteriological techniques, and anti-microbial susceptibility testing was done by Kirby Bauer's disk diffusion method. Extended-spectrum *β*-lactamase (ES*β*L) and carbapenemase production were confirmed by MASTTM D68C and MASTTM D73C combination disc sets, respectively. Chi-square and odds ratios were calculated.

**Results:**

Overall, 69.2% (27/39) of sputum samples were confirmed to be culture-positive. A total of 32 bacterial isolates with 78.1% (25/32) Gram-negative and 21.9% (7/32) Gram-positive bacteria were identified. The predominant bacterial isolates were *Pseudomonas aeruginosa* 21.9% (7/32), *Klebsiella pneumoniae* 18.75% (6/32), and *Staphylococcus aureus* 15.62% (7/32). Overall, 30 (93.8%) of the isolates were multidrug-resistant (MDR). About 48% (12/25) and 8 (32%)of gram negative bacterial isolates were ESBL betalatemase and OXA-48 carbapenemase producers, respectively. Having two or more exacerbation experiences in the previous year were found to be important determinants of the sputum culture positivity.

**Conclusions:**

High rates of MDR, ESBL, and carbapenemase producer bacteria were isolated from patients with AECOPD. Empiric antibiotic therapy should consider the prevalence of antibiotic-resistant pathogens and the factor that may increase the occurrence of MDR bacterial pathogens.

## 1. Introduction

Chronic obstructive pulmonary disease (COPD) is a major cause of chronic morbidity and mortality worldwide. Acute exacerbation is a common problem during the natural course of COPD, which is characterized by an increase in the patient's daily symptoms of dyspnea, cough, and/or sputum beyond normal day-to-day variability and severe enough to require a change in management [1, 2]. Frequent exacerbations are associated with an accelerated decline of lung function, reduced physical activity, poorer quality of life, and an increased risk for mortality [3]. According to the World Health Organization, COPD will become the third most common cause of death globally by 2030 and will be the fifth most economically burdensome disease due to frequent acute exacerbation [4].

The most common cause of acute exacerbations of COPD (AECOPD) is an infection of the tracheobronchial tree and air pollution. As many as one-third of AECOPD causes are never identified. The microbial etiology of AECOPD includes bacteria and viruses with more than 50% of cases being caused by bacterial infection [1]. The predominant causes of bacterial pathogens isolated from patients with exacerbation of COPD are *Haemophilus influenzae, Streptococcus pneumoniae,* and *Moraxella catarrhalis* [5, 6]. Other less commonly isolated potential pathogens are *Staphylococcus aureus*, *Pseudomonas aeruginosa*, and Gram-negative *Enterobacteriaceae* [6].

The bacterial etiologies of AECOPD keep changing from time to time and the choice of antimicrobial depends upon on local prevalence of bacterial etiologies and their resistance pattern [7]. Early diagnosis and knowledge of the predominant bacterial etiologies and antimicrobial resistance patterns will also help to correct treatment protocol for the management of AECOPD [8, 9]. To our knowledge, there is limited data about the bacteriological profile and their susceptibility pattern in AECOPD patients in Ethiopia. Therefore, this study was conducted to determine bacterial etiologies and their antibiotic resistance patterns among hospitalized patients with AECOPD at Jimma Medical Center (JMC).

## 2. Methods

### 2.1. Study Design and Setting

A cross-sectional study was conducted from March to October 2019 at Jimma Medical Center which is 354 km far from the capital and the only tertiary teaching hospital in the Southwestern part of the country. It has a capacity of 800 beds and serves a catchment population of over 20 million people.

### 2.2. Study Participants

All spirometry confirmed COPD patients admitted with AECOPD in the medical ward of JUMC during the study period were enrolled.

### 2.3. Study Variables

The list of study varibles is as follows:

### 2.4. Sample Size and Sampling Technique

#### 2.4.1. Sample Size

All patients admitted with AECOPD during eight months study period were included.

#### 2.4.2. Sampling Technique

Nonprobability consecutive sampling technique was used.

#### 2.4.3. Inclusion Criteria

All diagnosed patients with COPD according to the Global Initiative for Chronic Obstructive Lung Disease (GOLD) guideline 2022 and all AECOPD patients diagnosed by the concerned clinician depending upon the presence of two of the following symptoms having acute exacerbation with increased dyspnea, increased sputum volume, increased sputum purulence, and adequate sputum sample based on Bartlett's criteria [10, 11].

#### 2.4.4. Exclusion Criteria

All patients with known cases of tuberculosis, pneumonia, and asthma with evidence of either clinically or radiologically and patients, who were unable to participate, were excluded from the study.

### 2.5. Data Collection

Socio-demographic characteristics and clinical and behavioral factors of the patient were collected by face-to-face administered structured questionnaires using study variables listed in Table 1. A pretest was done to validate the questionnaire and required modification was done accordingly. Expectorated sputum specimens were collected in a sterile sputum cup (falcon tube) by standard collection procedures after ordering them to rinse their mouth with water. Patients were instructed to take a deep breath, hold it shortly, and then cough vigorously to produce quality sputum.

### 2.6. Sputum Processing

The sputum sample was transported to the Department of Medical Microbiology laboratory where the bacteriological analysis was performed. The quality of expectorated sputum samples was assessed by Bartlett's scoring method [11]. In addition, sputa were screened for nonprescreened cases (34 cases) for pulmonary tuberculosis by using the Ziehl–Neelsen (ZN) staining technique and all of them were smear-negative for *Mycobacterium tuberculosis*.

### 2.7. Gram Stain

The specimens were subjected to microscopic examination for their physical appearance of the specimens and microscopic examination to determine the acceptability of the specimen for culture. Gram-stained smears were prepared from the most visually purulent portions of the sputum specimen. The quality of sputum was assessed by determining the numbers of squamous epithelial cells (SECs) and polymorphonuclear cells (PMN) in the gram-stained smear of the specimen through microscopic examination within the following categories: <10, 10–25, or >25 cells per representative (100x) low power fields (LPF). The presence of PMN was graded as 0, +1, and +2, whereas SECs were graded as −2, −1, and 0 after observing a minimum of 20 LPF. The scores were added and the specimen with 1 or greater than 1 score was considered an acceptable specimen, while sputum specimens with zero or fewer scores were classified as being nonacceptable, then the good quality sputum specimens were further processed [11].

### 2.8. Washing Technique

Accepted samples were mixed with physiological saline (1 : 10 vol), vortexed, and centrifuged for 10 minutes at 1500 rpm. An equal volume of N-acetyl-L cysteine was added to the pellet for homogenization and the mixture was incubated at 37°C for about 15 minutes [10].

### 2.9. Specimen Culture

Samples were accepted after microscopic evaluation and washing procedures, sputum was inoculated directly onto blood agar base (Accumix, Tulip Diagnostics Ltd, India), MacConkey agar (Oxoid Ltd, England), and chocolate agar (Accumix, Tulip Diagnostics Ltd, India) plates for bacterial isolation. Washed samples as described above were further diluted 1 : 10–1 : 1000 in sterile saline and 10 *μ*l aliquot from 1 : 100 and 1 : 1000 dilution was inoculated on sheep blood agar, MacConkey agar, and chocolate agar plates for quantitative evaluation [12]. The chocolate agar plate was incubated in an incubator (5–10% CO_2_) at 37°C for 24–48 hours, while blood agar and MacConkey agar, were incubated in an aerobic atmosphere at 37°C for 24 hours. The suspicious colony was subcultured on suitable solid culture media for purification, thereafter further procedures were processed or preserved on appropriate media, and stored in a refrigerator (4°C) for subsequent analysis as necessary. Isolated micro-organisms were considered significant and accepted as causative pathogens only if they reached a count of 10^6^ CFU/mL [13].

### 2.10. Identification of Isolated Organisms

The identification of isolates was accomplished using the standard microbiological techniques, which involved morphological colony studies, Gram staining, and a battery of biochemical tests like Catalase test, Oxidase test, Indole, Citrate utilization test, Urease production, Hydrogen sulfide production, Sugar fermentation test, Coagulase test, and Optochin sensitivity tests were done [14].

### 2.11. Antimicrobial Susceptibility Test

The antimicrobial susceptibility pattern of isolates was done using the Kirby Bauer disk diffusion method on Mueller Hinton agar (Oxoid Ltd, England) for Penicillin (10 *μ*g), Cefoxitin (30 *μ*g), Clindamycin (2 *μ*g), and Erythromycin (15 *μ*g) for Gram-positive bacteria only. Tetracycline (30 *μ*g), Ampicillin (10 *μ*g), Chloramphenicol (30 *μ*g), Gentamycin (10 *μ*g), Ciprofloxacin (5 *μ*g), Trimethoprim-sulphamethoxazole (1.25 g), Ceftriaxone (30 *μ*g), Amoxicillin-clavulanic acid (10 *μ*g), and Cefepime (30 *μ*g) for both Gram-positives and Gram-negatives but Amikacin (30 *μ*g), Ceftazidime (30 *μ*g), Tobramycin (10 *μ*g), and Meropenem (10 *μ*g) for Gram-negative bacteria only according to Clinical Laboratory Standards Institute guidelines [15].

### 2.12. Extended-Spectrum Beta-Lactamases (ESBL), AmpC, and Carbapenemase Detection

Isolates were tested for ESBL and carbapenemase production using MAST™ D68C and D73C combination disc sets, respectively. After overnight incubation at 35–37°C, the zone of inhibition was measured and recorded on an excel sheet and then transported to Mast group ESBL/AmpC and CARBA plus calculator spreadsheet (Mast Group, UK) and interpreted as ESBL or/and AmpC and carbapenemase positive or negative accordingly.

### 2.13. Quality Control

The quality of the specimen was checked based on Bartlett's acceptance and rejection criteria. Furthermore, to decrease the heavy growth of commensal organisms from sputum culture, the sputum washing technique and quantitative culture of sputum were used [10, 12]. American Type Culture Collection (ATCC) control strains, *Pseudomonas aeruginosa*-ATCC 27853, *E. coli* ATCC 25922, *and S. aureus* ATCC 25923, were used according to CLSI guidelines [15]. ESBL positive ATCC 700603 *Klebsiella pneumoniae* and ESBL negative *E. coli* ATCC 25922 control strains were also used as a control for ESBL detection.

### 2.14. Statistical Analysis

After assuring the completeness of the data, it was entered in EpiData version 3.1 and exported to SPSS version 23 Software for analysis. Descriptive statistics were calculated and the results were presented in tables and graphs. Chi-square test and odds ratios with corresponding confidence intervals were calculated to ascertain the association between patients' demographic and clinical characteristics with outcomes. *p* values ≤0.05 were considered statistically significant.

### 2.15. Ethical Considerations

Before the commencement of the study, ethical approval was obtained from the institutional review board (IRB) of Jimma University with its reference number IHRPGD/565/2019. Written informed consent was obtained from the study participants or their guardians. Confidentiality of individual patients' information was maintained during data collection, analysis, and interpretation. The laboratory results of the study participant were reported to their treating physicians.

## 3. Results

### 3.1. Demographic and Clinical Characteristics

In this study, a total of 39 study participants were enrolled. Of these, 31 (79.5%) of them were males. The median age of patients was 65 years (interquartile range 25) with a maximum of the patients 38.5% in the age group 55−64 years. The majority of patients belonged to rural areas 25 (64.1%) and 19 (48.7%) of patients were farmers. History of tobacco smoking and biomass fuel-smoking exposure was reported in 28 (71.8%) and 15 (38.5%) patients, respectively. Of the total, 92.6% of patients had used antibiotics within the past three months before obtaining sputum samples; in addition, 64.2% and 43.6% of patients were inhalant steroid and systemic steroid users before data collection, respectively, (Table 2).

Based on spirometry results, AECOPD patients were graded depending upon the severity of airflow obstruction into mild, moderate, severe, and very severe (GOLD 2022). Out of 39 patients included in the study, the majority of patients 15 (38.5) and 11 (28.2%) had very severe and severe obstruction, and the remaining eight (20.5%) and five (12.8%) patients had mild and moderate obstruction, respectively, (Table 2).

### 3.2. Bacterial Profile among Patients with Acute Exacerbation of Chronic Obstructive Pulmonary Disease (AECOPD) Patients

The prevalence of potentially pathogenic bacterial isolates was 69.2%, (95% CI; 54–84). Of 27 patients, a total of 32 bacteria were isolated predominated by Gram-negative bacterial isolates 25 (78.1%). The commonest bacteria isolated from the specimens were *Pseudomonas aeruginosa* (*n* = 7, 21.9%) followed by *K. pneumoniae* (*n* = 6, 18.75%), S. aureus (*n* = 5, 15.62%), Escherichia coli (*n* = 4, 12.5%), Acinetobacter spp. (*n* = *4, 12.5%*), Proteus spp. (*n* = 3, 9.37%), S. pneumoniae (*n* = 2, 6.25%), and H. influenizae (*n* = 1, 3.12%) (Table 3). Out of 32 isolates, mixed bacterial isolates were revealed in 5 (13%) study participants. The most frequent coinfection occurred by K. pneumoniae or S. aureus with other bacteria in 18.5% of culture-positive cases for bacterial pathogens.

### 3.3. Antibacterial Resistance Patterns of the Isolates

Out of the tested antibiotics for Gram-negative bacteria were resistant to (100%) ampicillin, (96%) cefuroxime, and (88%) ceftriaxone (Table 4). Similarly, Gram-positive isolates were resistant to (85.7%) penicillin and tetracycline, (71.4%) azithromycin, and erythromycin. In addition, 80% of isolated S. *aureus* was Methicillin-resistant (Table 5). On the other hand, most of the Gram-negative isolates were relatively less resistant to meropenem (12%), amikacin (20.8%), gentamicin, tobramycin (29.2%), and ciprofloxacin (44%) (Table 4).

### 3.4. Prevalence of ESBL and Carbapenemase-Producing Gram-Negative Isolates

Of the 25 GNB isolates, 12 (48%), 6 (24%), and 7 (28%) were found ESBL, AmpC, and OXA-48 carbapenemase producers, respectively. Specifically, the prevalence of the ESBL-producing Klebsiella spp., P. aeruginosa, E. coli, and *Proteus spp.* were 4 (66.7%), 2 (28.6%), 3 (75%), and 2 (66.7%), respectively. In addition, 3(75%), 3(50%), and 2(50%) of *Acinetobacter* spp, P. *aeruginosa, and* E. *coli* isolates were OXA-48 carbapenemases producers, respectively (Table 6).

### 3.5. Prevalence of Multidrug-Resistant Isolates

Overall, multidrug resistance was observed in 30 (93.8%) of the isolates. Of these, 7 (100%) of P. aeruginosa, 6 (100%) of Klebsiella spp., 4 (100%) of E. coli. 5 (100%) of S. aureus, 1 (50%) of S pnumoniae, 4 (100%) of Acinetobacter spp. 3 (100%) of Proteus spp. isolates were found to be MDR (Table 7).

### 3.6. Factor Associated with Sputum Culture Positivity Inpatient Admitted with Acute Exacerbation of Chronic Obstructive Pulmonary Disease (AECOPD) at JUMC, Southwest Ethiopia, 2019

Both bivariate and multivariable analyses were conducted for selected variables that are adequate for logistic regression analysis. For acute exacerbation of COPD relative significant association (*p* < 0.25) was found between being age ≥65 years, (COR 4.8 (95% CI: 1.04–22.10), *p* ≤ 0.044), having two or more exacerbations in the previous one year (COR 7.27 (95% CI: 1.33–39.86), *p* ≤ 0.022) and systemic steroid use, (COR 6.25 (95% CI: 0.14–34.12), *p* ≤ 0.034) and sputum culture positivity (Table 8). In multivariable analysis, the only variable, having two or more exacerbations in the previous year (AOR 6.59 (95% CI: 1.06–38.73), *p* ≤ 0.037) was found to have a statistically significant association with positive sputum culture for the bacterial pathogen (Table 8).

### 3.7. Treatment and Inpatient Mortality

In the present study, 36 (92.3%) of study participants were engaged in antibiotics within three months before they enrolled in this study (Table 2). The most commonly used antibiotic was the third-generation cephalosporin (ceftriaxone) and azithromycin. The utilization rate of ceftriaxone among our study participants was 89.7% from isolated organisms. However, 78.6% of naturally susceptible Gram-negative isolates were found resistant to ceftriaxone. On the other hand, inpatient mortality of AECOPD patients was 8(20.5%) (Table 2). Of these deceased cases, 4(50%) and 3(37.5%) case mortality was attributed to multidrug-resistant MDR *P. aeruginosa* and *Acinetobacter* spp. induced infections, respectively.

## 4. Discussion

The acute exacerbation of the chronic obstructive pulmonary disease is a major reason for health care utilization including hospitalizations and the principal cause of death is usually secondary to bacterial infections [17]. In the present study, thirty-nine patients with AECOPD were included; with a mean age of 64.74 ± 11.37 years (median age of 65 years (interquartile range 20)) and the maximum of the patients (38.5%) in the age group 55−65 years (Table 2). This observation is comparable with other studies reported from India 40% (55–65 years) [18] and Indonesia 42% (55–65 years) [19] with a predominance of males. Although tobacco-smoking history was more prevalent in male patients, exposure to biomass fuel smoke was rife in homemaker females with a 38.5% prevalence among total study participants (Table 2). This is similar to a study report from Egypt; exposure to indoor air pollination was more common in females than nonsmokers [20]. This observation might be because smoking and air pollution are responsible for the decrease in mucociliary clearance that leads to increased bacterial colonization that can give rise to increased airway inflammation and as a result exacerbations [21]. In addition, male predominance could be tobacco-smoking habits is more common among males than females in our country, which is one of the major predisposing factors for the development of COPD.

In the present study, the overall prevalence of bacterial infection was 69.2 (95% CI; 54–84), which is comparable with other studies reported from Egypt 71% [20, 22], India 73.65% [23], Indonesia 71.2% [19], and Bangladesh 65.21% [24]. While a lower rate was reported in other studies from India 42.9% [25] and 47.22% [4] than the current study finding. On the other hand, a higher rate was reported from Egypt 94% [16]. Gram-negative bacterial isolate was predominating, 78.1% in this study. This finding was comparable with studies reported from India 77.78% [25]and Serbia 83% [26] account for Gram-negative bacteria. On the other hand, in other studies in Egypt, Gram-positive isolates account for 80% [20, 22]. The discrepancy in the prevalence of bacterial pathogens theses may be related to largely the type of studied participants. This study was done on hospitalized patients due to exacerbation of COPD, where these populations are more prone to infection with Gram negatives, whereas predominance of Gram-positive was more common in community-based outpatients [4, 27]. In the current study, single and mixed bacterial infections were isolated from 56.4% to 12.8% of the study population. This result is almost comparable with the study conducted in Serbia, coinfections were presented in 9.57% of the patients [26]. Having experienced two or more exacerbations in the previous year (AOR 6.6 (95% CI: 1.06–38.73)), (*p* ≤ 0.037) was found an independent factor associated with culture positivity. This is in line with studies that identified frequent exacerbation as an independent factor associated with culture positivity [4, 28]. Frequent exacerbations are associated with a faster decline in lung function. This decline in lung function favors colonization and/or infection with the bacterial pathogen by failure in host immunity, such as epithelial cell damage, mucous hypersecretion and inflammatory cell infiltrates, which makes them susceptible to frequent exacerbation [29].

When we come to specific bacterial isolates, in this study, *Pseudomonas aeruginosa* and K. pneumoniae were the predominant bacterial isolates as reported in similar studies from Egypt [16], Tunisia [30], and India [18]. On the other hand, to a study report from India [31], *S. pneumoniae* and *H. influenza* or methicillin-resistant *Staphylococcus aureus* (MRSA) were frequently isolated organisms. In addition, reports especially on western population micro-organisms in AECOPD patients were *S. pneumoniae* followed by *H. influenzae* or *S. aureus* then Gram-negative bacteria [5, 6].

The variability in types of bacterial pathogens may be related to the type of studied participants. In hospitalized and severely ill patients*, P. aeruginosa* and other Gram negatives have also been reported whereas predominance of *S. pneumoniae* and *H. influenzae* or *S. aureus* were more common in community-based outpatients [4–6, 27]. Besides, this difference can also be explained by the differences in variation in the spread of respiratory infections between populations and countries, depending on a difference in geography, climate, and socioeconomic conditions as well as it might be due to misuse of antibiotics. Furthermore, the difference in culture positivity and distribution of micro-organisms could be the result of the different methods of collection, transportation time and the number of organisms present in the sample, and the nature of the sputum [4, 6, 27].

This study showed that 30 (93.8%) of the bacterial isolates were MDR. Most frequently isolated organism, P. aeruginosa resistance rate was 100% for ceftazidime, cefepime, cotrimoxazole, chloramphenicol, tetracycline, and cefuroxime. While (100%) meropenem, and (85.7%) Ciprofloxacin were the most effective antibiotics for P. aeruginosa. Similarly, the second most isolated organism, K. pneumonia also showed 100% resistance to ampicillin and cefuroxime, but has good susceptibility to some drugs like meropenem, amikacin, gentamicin, amoxicillin/clavulanate, and ciprofloxacin. Similar antibiotic susceptibility patterns were reported in different studies [4, 18, 23, 25]. Regarding the Gram-positive bacteria, 4 (80%) of total *S. aureus* isolates showed a high rate of methicillin resistance, which is comparable with Indian studies [18, 23]. Furthermore, the prevalence of ESBL and OXA-48 carbapenemase producers 48% (12/25) and 8 (32%)of gram negative bacterial isolates, respectively; this could be due to repeated exposure to such beta-lactams drugs during frequent exacerbations [32, 33].

Even though guidelines recommend combination therapy with amoxicillin/clavulanate or cephalosporin and macrolide or doxycycline or macrolide with a respiratory fluoroquinolone for initial empirical treatment for hospitalized patients with acute exacerbation of COPD [1, 34], majority of the bacterial isolates (68–92%) in the currents were resistant to the listed drugs (Tables 4 and 5). This finding is comparable with the report in Egypt [3, 35] and India [36]. This may be due to the regular use of these antibiotics that have been prescribed frequently, which contribute to the development of drug resistance.

In this study, inpatient mortality of AECOPD patients was 20.5% with 87.5% mortality due to MDR P. aeruginosa and Acinetobacter species-induced infections (Table 2). In different studies reported from Egypt [16] and Serbia [26] mortality among COPD exacerbation patients was (50%) and (6.2%) with 100% P. *aeruginosa* induced infection, respectively. In this study, mortality was lower than reported mortality from Egypt and higher than mortality reported from Serbia. The reason could be a difference in the severity of the disease (about 40% of our study participants were at the last stage of COPD), sample size, and treatment options.

### 4.1. Limitation of the Study

This study has several limitations, first, the nature of the participants and antibiotic treatment before the diagnosis could be biased for pathogen frequencies identified in this study. Moreover, sputum samples were the specimen used in this study, which may be contaminated. To overcome this problem, we have used sputum validity criteria proposed by different researchers. The other limitation was the limited sample size, which did not permit us to perform a multivariable analysis for the identification of different exclusive factors associated with culture positivity. Although combinations of aminoglycosides and carbapenems were tested, other beta-lactams, beta-lactamase inhibitors, and newer fluoroquinolones, such as tigecycline, colistin, piperacillin/tazobactam, and levofloxacin, were not tested. In the last, the study site was limited to JUMC, where the data obtained might not be representative of the entire Ethiopian hospitalized adult population.

## 5. Conclusions

In the current study, a high rate of culture positivity was reported with P. aeruginosa, Klebsiella pneumoniae, and S. aureus the predominant bacterial isolates. Many isolated strains have shown a high rate of MDR pattern, beta-lactamase and carbapenemase production. Thus, empiric antibiotic therapy should consider the prevalence of MDR bacterial pathogens in AECOPD patients. Furthermore, periodic surveillance of etiologies of AECOPD and their anti-microbial susceptibility pattern is essential to improve the quality of care and contain anti-microbial resistance.

## Figures and Tables

**Table 1:** List of study variables.

	Variables	
Independent variables	Age	Number of exacerbation's in the previous year
	Sex	The severity of COPD
	Alcohol consumption	Biomass fuel exposure
	Residence	Comorbidities
	Smoking status	History of steroid/antibiotic use

Dependent variables	Culture result	
	Type of bacteria	
	Antibiotics resistance	

**Table 2:** Demographic and related clinical characteristics of patients with AECOPD at JUMC, southwest Ethiopia, 2019. (*n* = 39) in JUMC.

Variables		Total no. 39	Culture-positive cases, 27 (69.2%)
Age, year	45-54	4 (10.3)	1 (3.7)
	55–64	15 (38.5)	10 (37)
	65–74	10 (25.6)	7 (25.9)
	≥75	10 (25.6)	9 (33.4)

Sex	Female	8 (20.5)	7 (25.9)
	Male	31 (79.5)	20 (74.1)

Residence	Urban	14 (35.9)	9 (33.3)
	Rural	25 (64.1)	18 (66.7)

Tobacco smoking status	No	11 (28.2)	8 (29.6)
	Yes	28 (71.8)	19 (70.4)

Biomass fuel-smoking status	No	24 (61.5)	16 (59.3)
	Yes	15 (38.5)	11 (40.7)

No. of exacerbations in previous one year	≤1	18 (46.2)	8 (29.6)
	≥2	21 (53.8)	19 (70.4)

Inhalant steroid use	No	14 (35.9)	8 (29.6)
	Yes	25 (64.2)	19 (70.4)

Systemic steroid use	No	22 (56.4)	12 (44.4)
	Yes	17 (43.6)	15 (56.6)

Stage of COPD	Mild	5 (12.8)	3 (11.1)
	Moderate	8 (20.5)	5 (18.5)
	Sever	11 (28.2)	8 (29.6)
	Very severe	15 (38.5)	11 (40.7)

History of antibiotics usage	No	3 (7.7)	2 (7.4)
	Yes	36 (92.3)	25 (92.6)

Discharge outcome/died	No	31 (79.5)	19 (70.4)
	Yes	8 (20.5)	8 (29.6)

**Table 3:** Sputum bacteriology from acute exacerbation of chronic obstructive pulmonary disease patients at JUMC, southwest Ethiopia, 2019.

Gram-reaction	Isolated species	Frequency	Percentage (%)
Gram-positive bacteria	Staphylococci aureus	5	15.62
	Streptococcus pneumoniae	2	6.25

Gram-negative bacteria	Pseudomonas aeruginosa	7	21.9
	Klebsiella pneumoniae	6	18.75
	Escherichia coli	4	12.5
	Acinetobacter spp.	4	12.5
	Proteus spp.	3	9.37
	Haemophilus influenzae	1	3.12

**Table 4:** Antibiotic susceptibility pattern of Gram-negative bacterial pathogens to tested antibiotics in patients with AECOPD at JUMC, southwest Ethiopia, 2019.

Isolates	Antibiotic susceptibility pattern (%)													
	TOB	CN	AMK	CAZ	FEP	CRO	AMC	CAF	SXT	AMP	CXM	CIP	MRP	Doxy
K. pneumoniae [6]	33.3	33.3	33.3	83.3	66.7	83.3	33.3	66.7	83.3	100	100	50	0	83.3
E.coli [4]	25	25	25	75	75	75	75	50	100	100	100	50	25	75
Proteus spp. [3]	33.3	33.3	33.3	100	100	100	66.7	100	100	100	100	100	33.3	100
P. aeruginosa [7]	28.6	28.6	14.3	100	100	100	100	100	100	NT	NT	14.3	0	100
Acinetobacter spp. [4]	25	25	25	100	75	100	75	75	100	NT	NT	50	25	100
H. influenzae [1]	NT	NT	NT	0	0	0	0	0	0	0	0	0	0	100
Total [16]	29.2	29.2	33.3	88	80	88	68	76	92	100	96	44	12	92

TOB-tobramycin, CN-gentamycin, CAZ-ceftazidime, FEP-cefepime, AMC-amoxicillin-clavulanate, CRO-ceftriaxone, CIP-ciprofloxacin, MRP–meropenem, AMP-ampicillin, SXT-trimethoprim-us, Doxy-doxycycline, NT-not tested, and spp: species.

**Table 5:** Antibiotic susceptibility pattern of Gram-positive bacterial pathogens to tested antibiotics in patients with AECOPD at JUMC, southwest Ethiopia, 2019.

Isolate	Antibiotic resistance pattern (%)										
	P	ERY	CLD	VAN	SXT	CAF	OX	CIP	CN	AZM	DOXY
S. aureus [5]	100	80	60	NT	60	60	80	40	20	60	80
S. pneumoniae [2]	50	50	0	0	0	0	NT	NT	NT	100	50
Total	85.7	71.4	57.1		42.9	42.9			20	71.4	57.1

Key, P-Penicillin, CN-gentamycin, CAZ-ceftazidime, FEP-cefepime, CIP-ciprofloxacin, FOX-cefoxitin, SXT-trimethoprim-, Doxy-doxycycline, ERY-erythromycin, CLD-clindamycin, VAN-vancomycin, NT-not tested, and spp: species.

**Table 6:** Distributions of *β*-lactamases and carbapenemases in gram-negative bacteria isolated from patients with acute exacerbation of chronic obstructive pulmonary disease at JUMC, southwest Ethiopia, 2019.

Isolates	AmpC+	OXA-48+	ESBL+	ESBL and AmpC+	AmpC+ and OXA-48+	ESBL, AmpC and OXA-48 +
Acinetobacter spp [4]	1	3	1		1	
K. pneumoniae [6]	2	0	4			
P. aeruginosa [7]	1	3	2	1		
E.coli [4]	2	2	3			2
Proteus spp [3]	0	0	2			
	6 (24%)	8 (32%)	12 (48%)			

ESBL^+^: extended beta-lactamase positive; spp: species.

**Table 7:** Distribution of antibiotics resistance of bacterial isolates from acute exacerbation of chronic obstructive pulmonary disease patients to different antibiotic classes at JUMC, southwest Ethiopia, 2019.

Isolates	NO	Penicillins		3rd generation cephalosporin		4th generation cephalosporin	Aminoglycoside		Phenicols	Tetracycline	Folate pathway inhibitors	Amoxicillin + Calculates	Fluoroquinolone	Cephamycins	Carbapenems	Lincosamides	Macrolides
		P	Amp	CRO	CAZ	FEP	TOB	CN	CAF	DOXY	SXT	AMC	CIP	FOX	MERR	CLD	ERY
*K. pneumoniae*	6	—	6	5	5	4	1	1	4	4	5	2	2	—	0	—	—
*E. coli*	4	—	4	3	3	3	1	1	2	3	4	3	2	—	1	—	—
*Proteus spp.*	3	—	3	3	3	3	1	1	2	3	3	2	3	—	1	—	—
*P. aeruginosa*	7	—	—	7	7	7	3	3	7	7	7	7	1	—	0	—	—
*Acinetobacter spp.*	4	—	—	4	4	3	2	2	3	4	4	3	2	—	1	—	—
*H. influenza*	1	—	—	—	0	0	—	—	0	1	0	0	1	—	0	—	—
*S. aureus*	5	5	—	—	—	—	1	1	3	4	3	—	2	5	—	3	4
*S. pneumoniae*	2	1	—	—	—	—	—	—	0	1	0	—	—	—	—	0	1

CAZ-ceftazidime, FEP-cefepime, AMC-amoxicillin-clavulanate, CRO-ceftriaxone, MRP–meropenem, CAF-chloramphenicol, AMP-ampicillin, P-penicillin, TOB-tobramycin, CN-gentamycin, CIP-ciprofloxacin, FOX-cefoxitin, SXT-trimethoprim-sulfamethoxazole, Dox-doxycycline, ERY-erythromycin, CLD-clindamycin, and spp., species.

**Table 8:** Bivariate and multivariable analysis of factors associated with sputum culture positivity among patients with acute exacerbation of chronic obstructive pulmonary disease at JUMC, southwest, Ethiopia, 2019.

Variables		Culture positive		COR (95% CI)	*p* value	AOR (95% CI)	*p* value
		Yes *n* (%)	No *n* (%)				
Age, year	36–49						
	50–64	10 (52.6)	9 (47.4)				
	>65	16 (84.2)	3 (15.6)	4.8 (1.04–22.10)	0.044	3.7 (0.67–19.99)	0.134

Residence	Urban	9 (64.3)	5 (35.7)				
	Rural	18 (72)	7 (28)	1.4 (0.35–5.79)	0.617		

Inhalant steroid use	No	8 (57.1)	6 (42.9)				
	Yes	19 (76)	6 (24)	2 (0.50–7.99)	0.333		

Systemic steroid use	No	12 (5.6)	10 (45.4)				
	Yes	15 (88.2)	2 (11.7)	6.3 (1.14–34.12)	0.034	4.6 (0.75–27.69)	0.142

Exacerbation experience in the previous year	≤1	11 (40.7)	10 (83.3)				
	≥2	16 (59.3)	2 (16.7)	7.3 (1.33–39.86)	0.022	6.6 (1.06–38.73)	0.037^*∗*^

AECOPD: acute exacerbation of the chronic obstructive pulmonary disease, COR = crude odds ratio; CI = confidence interval; AOR = adjusted odds ratio; and ^*∗*^statically significant.

## Data Availability

The datasets used and/or analyzed during the current study are available from the corresponding author on reasonable request.

## Abbreviations

COPD: Chronic obstructive pulmonary disease
AECOPD: Acute exacerbation of chronic obstructive pulmonary disease
ESBL: Extended spectrum beta-lactamase
MDR: Multidrug resistance
JMC: Jimma medical center.
